# Difference of Farmers' Livelihood Capital before and after Rural Tourism Development

**DOI:** 10.1155/2022/4138220

**Published:** 2022-03-27

**Authors:** Xiao Yi, Tan Xixi, Pan Lu

**Affiliations:** ^1^College of Public Administration, Chongqing Technology and Business University, Chongqing 400067, China; ^2^College of Business Administration, Chongqing Normal University, Chongqing 400047, China

## Abstract

*Purpose*. To compare the differences in rural household livelihood capital before and after the development of rural tourism to derive factors that affect rural household livelihood capital. *Methodology*. This study establishes a household livelihood capital index system to determine the total livelihood capital owned by rural households. *Findings*. After the development of rural tourism, regardless of farmers participating in rural tourism or not participating in rural tourism, their livelihood capital has increased, but the growth rate of livelihood capital of farmers participating in rural tourism is significantly higher than that of non-participating farmers, especially social capital and financial capital. *Originality*. This study is based on the sustainable livelihood analysis framework developed by DFID, analyzes the characteristics of farmers' livelihood capital and livelihood activities, and discusses the differences of farmers' livelihood capital before and after rural tourism development.

## 1. Introduction

Rural tourism is an important part of China's rural development and tourism industry development in the new century and an important driving force to promote rural economic development. The development of rural tourism has an important impact on farmers, agriculture, and rural areas, among which the impact on farmers is the core. Therefore, it is of great theoretical value and practical significance to study rural tourism development on the stock and flow of farmers' livelihood assets, asset portfolio and conversion, and the ability to use assets. This can not only provide decision support for the development of rural tourism in the region but also play an important role in reducing the vulnerability of poor people and promoting economic development.

Rural tourism will change the reserve and combination of farmers' livelihood capital, for households with insufficient capital stock, insufficient flow, and weak transfer and mobility, and it is very likely to fall into poverty in the process of rural tourism development [1–3]. Rural tourism is not a simple external force, and the rural community is not static, but in a state of continuous change, and it is difficult to judge or distinguish whether the result of farmers' livelihood is caused by tourism or other modern forces. Therefore, it is necessary to have a comprehensive perspective on the differences of farmers' livelihood capital in the development of rural tourism. This study is based on the sustainable livelihood analysis framework developed by DFID, analyzes the characteristics of farmers' livelihood capital and livelihood activities, and discusses the differences of farmers' livelihood capital before and after rural tourism development. Then, it puts forward some policy suggestions to improve and perfect the development of regional rural tourism and promote the sustainable livelihood development of farmers.

Based on the SLA of the Department of International Development of the United Kingdom combined with the domestic and foreign scholars' research methods on the livelihood capital of farmers [4, 5], the paper divides the livelihood capital of farmers into natural capital (N), human capital (H), material capital (P), social capital (S), and financial capital (F). Natural capital (N) refers to the natural resource reserve owned by farmers, which is calculated by three indicators: per capita cultivated land, per capita forest land, and per capita garden land. Human capital (H) refers to the health status and skills and knowledge possessed by the workers in the family, that is, the ability and means of making a living possessed by the farmers [6, 7]. Specifically, it includes five indicators: the total population, the labor force engaged in tourism, other labor force population, non-labor force population, and the education level of the labor force population (including primary school and below, junior high school to technical secondary school, junior college, and undergraduate and above). Physical capital (P) mainly refers to the material equipment and public facilities used by farmers for life and production, including household equipment, per capita housing area, housing construction materials, and daily energy consumption. Social capital (S) refers to social resources that people can use to achieve their livelihood goals, including the number of neighborhood assistance, skills training times, the number of government institutions, endowment insurance amount, community organizations, and the number of minimum insurance. Financial capital (F) refers to the amount of funds or credit help that farmers can obtain in order to achieve their own production goals, including annual per capita income of households, types of income sources, annual per capita expenditure of households, access to credit opportunities, and other indicators [8–11].

## 2. Materials and Methods

### 2.1. Data Collection

This study is based on a thorough understanding of the development of rural tourism in Chongqing; combined with the list of leisure agriculture and rural tourism demonstration towns, demonstration villages (communities), and demonstration sites in Chongqing in 2019, Damu Township in Fuling District is selected as the research destination of this paper. On July 12, 2019, the research group organized members of the research group to conduct a household questionnaire survey on farmers in Damu Township. A total of 120 questionnaires were sent out and 112 were recovered, of which 108 were effective, with an effective recovery rate of 90%.

### 2.2. Determination of Livelihood Capital Weight

According to the difference degree of various indicators, the objective entropy method is used to assign the weight of various livelihood capital, and the information entropy is used to determine the weight of each indicator (Table 1).

### 2.3. Measurement of Farmers' Livelihood Capital

According to the standardization of various livelihood capital of farmers and the determination of the weight of various livelihood capital by the entropy method, the paper calculates the livelihood capital of farmers by using each weight. The calculation formula of the livelihood capital *T* of farmers is as follows [12]:

(1) $$\begin{array}{c} T=\sum_{t=1}^{5}\sum_{j=1}^{n}W_{ij}I_{ij}, \end{array}$$

where *W*_*ij*_ is the weight of the *j*-th evaluation index of class I livelihood capital. In order to make the data more accurate, the weight adopted in this study is unified as the weight calculated by the present value of farmers' livelihood capital. *I*_*ij*_ is the standardized value of the *j*-th evaluation index of the I-type livelihood capital. In this paper, the calculation of the overall livelihood capital of farmers is to take the average value of various livelihood capital and then use the weight and the proportion of the quantitative treatment [13–15].

## 3. Results and Discussion

### 3.1. The Total Change Characteristics of Farmers' Livelihood Capital before and after Rural Tourism Development

From the calculation results of farmers' livelihood capital (Table 1), it can be seen that the total value of farmers' livelihood capital has increased on the whole, from 0.1795 before the development of rural tourism to 0.1877 after the development of rural tourism. But the order and change of these five kinds of specific farmers' livelihood capital are different. After the development of rural tourism, the human capital and natural capital of farmers have decreased, financial capital and material capital have increased, and social capital has hardly changed. It can be seen that after the development of rural tourism, the composition of farmers' livelihood capital has become more reasonable, and the proportion of various types of livelihood capital has gradually become more balanced.

### 3.2. Comparison of the Difference in the Livelihood Capital of Participating Farmers before and after the Development of Rural Tourism

The livelihood capital of farmers participating in rural tourism has changed significantly. Among the 69 rural households participating in rural tourism, 46 households had an increase in their total livelihood capital, accounting for 66.67% of the total participating farmers, and 23 households had a decrease in their total livelihood capital (Table 1).

Among participating farmers, the minimum value of rural households livelihood capital increased from 0.0444 to the current 0.0889, the maximum value of rural households livelihood capital decreased from 0.4386 to 0.3929, and the average value of rural households' total livelihood capital increased from 0.1861 to 0.2180. It can be seen that the minimum and average values of total livelihood capital of participating farmers have increased, while the maximum value of total livelihood capital has decreased, which indicates that the overall livelihood capital of participating farmers has increased. Also, the gap between participating farmers is gradually narrowing.

**Figure 1:**
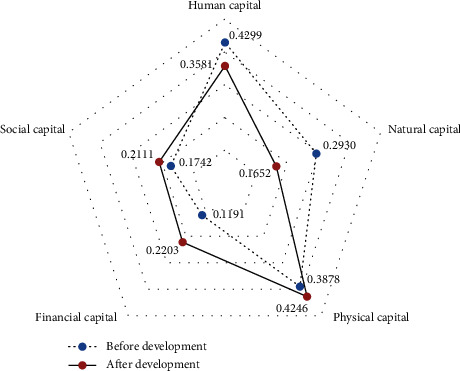
Comparison of livelihood capital of participating farmers before and after the development of rural tourism.

Specifically, financial capital and natural capital have changed the most. Financial capital increased from 0.1191 before tourism development to 0.2203 after tourism development, and natural capital decreased from 0.2930 before tourism development to 0.1652 after tourism development. In addition, human capital also decreased, from 0.4299 to 0.3581; physical capital and social capital increased, social capital rose from 0.1742 to 0.2111, and material capital rose from 0.3878 to 0.4246. This shows that in the livelihood capital of farmers participating in rural tourism, the proportion of their livelihood capital structure has changed, and the importance and proportion of the three types of livelihood capital, financial capital, social capital, and physical capital, have increased. Natural capital and human capital have decreased. Human capital has dropped from the original number one to the current number two; natural capital has fallen from the original number two to the current number four. This shows that after the development of rural tourism, farmers' natural the proportion of total capital have dropped to a greater extent. From this change, it can be seen that the proportion of various types of livelihood capital of rural households participating in rural tourism gradually tends to balance (see Figure 1 for details).

### 3.3. Comparison of the Difference in the Livelihood Capital of Non-Participating Rural Households before and after the Development of Rural Tourism

Among the 39 households that did not participate in rural tourism, 12 households had an increase in the total value of their livelihood capital, accounting for 30.77% of the total non-participating households; the total value of livelihood capital decreased by 27 rural households, accounting for 69.23% of the total non-participating rural households. Among the non-participating households, the minimum value of the total value of household livelihood capital dropped from 0.0610 to 0.0450, and the maximum value of household livelihood capital increased from 0.3114 to 0.3542, and the average value of the total value of rural household livelihood capital has dropped from 0.1679 to 0.1341 now. It can be seen that the total value of livelihood capital of non-participating farmers has also declined, and the gap in total livelihood capital of non-participating farmers has gradually widened (Table 1).

**Figure 2:**
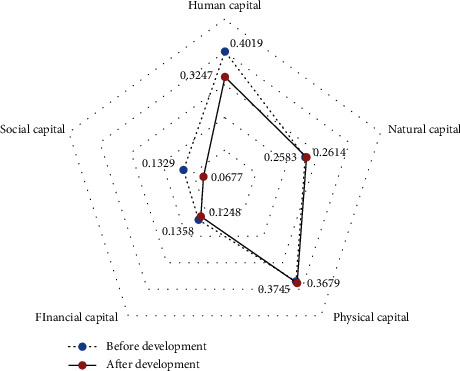
Comparison of livelihood capital of non-participating farmers before and after the development of rural tourism.

Specifically, the biggest changes are in social capital and human capital, both of which have decreased, while the other three types of capital have changed slightly. Natural capital and physical capital have all increased, while financial capital has decreased. It shows that human capital, natural capital, and material capital have always been very important among farmers who have not participated in the development of rural tourism. Human capital dropped from 0.4019 to 0.3247. Human capital is mainly manifested in the number of labor force of the farmer households and the degree of education of the labor force, which is the most direct factor influencing the choice of livelihood strategies for farmer households. The reason why their human capital will decrease is mainly because, compared with the development of rural tourism, although their labor education has increased, their labor population is gradually decreasing; natural capital increased from 0.2583 to 0.2614, with little change. Natural capital mainly refers to the amount of farmland, woodland, and gardens owned by farmers. Among the farmers who have not participated in rural tourism, the vast majority are farmers who are older or far away from the scenic area. Natural capital is particularly important to them, so their natural capital has not changed much before and after the development of rural tourism; social capital has changed significantly, from 0.1329 to 0.0677, mainly due to fewer and fewer farmers who are willing to stay in the countryside than before the development of rural tourism, and fewer contacts between farmers neighbors and relatives of farmers. Social capital has shown a clear downward trend. In the physical capital, household equipment and housing structure have increased, but the per capita housing area has decreased, and overall physical capital has increased (see Figure 2 for details).

### 3.4. Comparison of the Difference in Livelihood Capital between Participating Farmers and Non-Participating Farmers before and after the Development of Rural Tourism

Before the development of rural tourism, although the livelihood capital of farmers participating in rural tourism was generally higher than that of farmers not participating in rural tourism, the difference in livelihood capital of the two types of rural households was not significant. The top three rankings of their livelihood capital are human capital > physical capital > natural capital, which shows that before the development of rural tourism, the livelihood capital of rural households is mainly human capital, material capital, and natural capital; the latter two are different. The order of farmers participating in rural tourism is as follows: social capital > financial capital, and the order of farmers not participating in rural tourism is as follows: financial capital > social capital. This indicates that social capital may be a factor that affects farmers' participation in rural tourism.

After the development of rural tourism, farmers' livelihood capital has undergone major changes. There is a big gap between the livelihood capital of rural households participating in rural tourism and that of non-participating rural households. The average value of livelihood capital of participating rural households is 0.2180, and the average value of total livelihood capital of non-participating rural households is 0.1341. The order of various types of livelihood capital is also quite different. Among them, the order of livelihood capital of participating farmers is physical capital > human capital > financial capital > social capital > natural capital; the order of livelihood capital of non-participating farmers is physical capital > human capital > natural capital > financial capital > human capital. This shows that after the development of rural tourism, the financial capital, social capital, and material capital of rural households participating in rural tourism have increased significantly, and natural capital has decreased significantly compared with non-participating rural households. In addition, as a whole, the distribution of various types of capital of participating farmers is more balanced, which indicates that the sources of livelihood capital of participating farmers are more diverse and rich, while the sources of livelihood capital of non-participating farmers still rely on natural resources to a greater extent.

## 4. Conclusion

This study selected Damu Township, Fuling District, Chongqing City, as the research area, established a household livelihood capital index system to determine the total livelihood capital owned by rural households, and then compared the differences in rural household livelihood capital before and after the development of rural tourism to derive factors that affect rural household livelihood capital. The results show the following:

1. The minimum and average values of total livelihood capital of participating farmers have increased, while the maximum value of total livelihood capital has decreased. Specifically, financial capital and natural capital have changed the most.
2. The total value of livelihood capital of non-participating farmers has also declined, and the gap in total livelihood capital of non-participating farmers has gradually widened. Specifically, the biggest changes are in social capital and human capital, both of which have decreased, while the other three types of capital have changed slightly.
3. After the development of rural tourism, regardless of farmers participating in rural tourism or not participating in rural tourism, their livelihood capital has increased, but the growth rate of livelihood capital of farmers participating in rural tourism is significantly higher than that of non-participating farmers, especially social capital and financial capital.

## Figures and Tables

**Table 1:** The evaluation index system of farmers' livelihood capital.

Primary indicator	Measurement index	Weight	Measurement index value		Assignment
			Before development	After development	
Human capital	Family size	0.0069	0.0035	0.0034	Household registered residence population
	Family labor force as a whole	0.0149	0.0055	0.0034	It refers to the number of people aged 18 and above and 60 and below who can use their own ability to obtain the main source of income
	Education level of labor force	0.0154	0.0053	0.0050	Taking the family labor force aged 18 and above and under 60 as the target
Natural capital	Cultivated land per capita	0.0533	0.0175	0.0126	Per capita arable land (hm^2^)
	Woodland per capita	0.0179	0.0045	0.0055	Per capita arable land (hm^2^)
	Garden land per capita	0.0788	0.0201	0.0119	Per capita arable land (hm^2^)
Physical capital	Household equipment	0.0031	0.0010	0.0019	Household equipment index is measured by the number of household equipment owned by farmers
	Housing area per capita	0.0169	0.0053	0.0041	Per capita housing area (m^2^)
	Materials for housing construction	0.0052	0.0032	0.0042	According to the building structure of farmers' housing, the value is assigned
Financial capital	Wage income	0.0453	0.0038	0.0059	Wage income refers to the total labor remuneration that the employed people get through various ways
	Operating income	0.0517	0.0111	0.0102	It refers to the income of farmers through regular production and operation activities
	Property income	0.0780	0.0028	0.0141	It refers to the income obtained by farmers from real estate (such as houses, vehicles, collectibles, and so on) and movable property (such as bank deposits and securities) owned by families
	Other income	0.1024	0.0033	0.0090	Other income mainly includes government subsidies and other transfer income
	Total per capita income	0.0197	0.0020	0.0033	The total income of all members of the family
	Total expenditure per capita	0.0012	0.0011	0.0011	The sum of the expenses of all members of the family
	Per capita net income	0.0112	0.0021	0.0034	Family per capita net income = total per capita income-total per capita expenditure
	Types of help seeking methods	0.0550	0.0194	0.0207	Family help methods include bank or credit union loans, usury, borrowing money from relatives and friends, government, and social assistance
Social capital	Number of neighborhood help	0.0844	0.0419	0.0212	Community neighborhood assistance includes financial support (funds donation and interest-free loans), policy support (preferential, support policies, etc.), technical support (strategy, knowledge, etc.), and human support (labor)
	Skill training times	0.0578	0.0011	0.0166	Skills training includes various training such as farmer production and operation training, agricultural planting training, and farmer employment training
	Serving in government institutions	0.1597	0.0068	0.0156	Serving in government institutions
	Number of community organizations	0.1243	0.0181	0.0145	Community organizations include community cooperative organizations, agricultural collective economic organizations, agricultural production cooperatives, associations of famous people, etc.
Total		1	0.1794	0.1876	

## Data Availability

The datasets used and/or analyzed during the current study are available from the corresponding author on reasonable request.
